# High-pressure protein crystallography of hen egg-white lysozyme

**DOI:** 10.1107/S1399004715000292

**Published:** 2015-03-26

**Authors:** Hiroyuki Yamada, Takayuki Nagae, Nobuhisa Watanabe

**Affiliations:** a Department of Biotechnology, Graduate School of Engineering, Nagoya University, Chikusa, Nagoya, Aichi 464-8603, Japan; b Synchrotron Radiation Research Center, Nagoya University, Chikusa, Nagoya, Aichi 464-8603, Japan

**Keywords:** hen egg-white lysozyme, high pressure

## Abstract

Crystal structures of hen egg-white lysozyme (HEWL) determined under pressures ranging from ambient pressure to 950 MPa are presented. From 0.1 to 710 MPa, the molecular and internal cavity volumes are monotonically compressed. However, from 710 to 890 MPa the internal cavity volume remains almost constant. Moreover, as the pressure increases to 950 MPa, the tetragonal crystal of HEWL undergoes a phase transition from *P*4_3_2_1_2 to *P*4_3_. Under high pressure, the crystal structure of the enzyme undergoes several local and global changes accompanied by changes in hydration structure. For example, water molecules penetrate into an internal cavity neighbouring the active site and induce an alternate conformation of one of the catalytic residues, Glu35. These phenomena have not been detected by conventional X-ray crystal structure analysis and might play an important role in the catalytic activity of HEWL.

## Abstract 2

The crystal structure of hen egg-white lysozyme (HEWL) was analyzed under pressures of up to 950 MPa. The high pressure modified the conformation of the molecule and induced a novel phase transition in the tetragonal crystal of HEWL.

## 1. Introduction   

High pressure induces structural changes in proteins, such as the dissociation of oligomers, conformational changes and denaturation. For this reason, high-pressure structural analysis has been widely used to study important characteristics of proteins such as their stability, folding and aggregation (see, for example, Balny, 2006 ▶). Because the effect of pressure on protein structures helps us to understand their dynamics, structural studies based on high-pressure nuclear magnetic resonance (NMR) and X-ray crystallography have recently evolved (Cioni & Gabellieri, 2011 ▶; Fourme *et al.*, 2012 ▶). In the development of high-pressure X-ray protein crystallography (HPPX), the introduction of the diamond anvil cell (DAC) was a key step (Katrusiak & Dauter, 1996 ▶; Fourme *et al.*, 2001 ▶; Girard *et al.*, 2007 ▶). Using a DAC, the useful pressure range was extended by almost an order of magnitude relative to beryllium cells, which had previously been used to study protein crystals at pressures of less than 100 MPa. For example, the use of DACs permitted structural studies to be made of bovine copper zinc superoxide dismutase (SOD) at 1 GPa (Fourme *et al.*, 2001 ▶) and of Cowpea mosaic virus at 330 MPa (Girard *et al.*, 2005 ▶). Our group also developed a HPPX environment at the Photon Factory, Japan (Chavas *et al.*, 2013 ▶), with which we studied the pressure-induced structural changes of 3-isopropylmalate dehydrogenase (IPMDH) up to 650 MPa (Nagae *et al.*, 2012 ▶). In particular, the results for SOD and IPMDH show that the pressure-induced structural changes observed by HPPX are directly related to protein activity or function.

Compared with NMR, HPPX is a superior method because it is capable of directly detecting pressure-induced changes in hydration structure. Collins *et al.* (2005 ▶) reported water filling of the nonpolar interior cavity of L99A mutant T4 lysozyme. As the initial steps of pressure denaturation, Nagae *et al.* (2012 ▶) also observed water penetration into the hydrophobic cavity at the dimer interface and a cleft on the surface of IPMDH. To further study high-pressure effects in proteins, including pressure-induced changes in hydration structure, we used HPPX to study the well known tetragonal crystal of HEWL. The first high-pressure studies of the crystal structure of HEWL were made at 100 MPa using a beryllium cell (Kundrot & Richards, 1987 ▶). A DAC was then used to measure the compressibility of orthorhombic crystals up to 1.0 GPa (Katrusiak & Dauter, 1996 ▶), and a higher pressure structure of HEWL at 820 MPa was determined using a DAC (Fourme *et al.*, 2001 ▶, 2003 ▶). To our knowledge, however, no details of the high-pressure structure of HEWL have yet been reported.

HEWL is one of the best characterized enzymes. Its reaction mechanism was discussed for many years, and was settled by evidence for a glycosyl-enzyme covalent intermediate at Asp52 (Vocadlo *et al.*, 2001 ▶). However, basic questions remain for the other catalytic residue, Glu35. The protonated state of the carboxyl group of Glu35 or its abnormally elevated p*K*_a_ is known to be important to its catalytic reaction (Phillips, 1967 ▶). The p*K*_a_ of Glu35 has been experimentally confirmed to range from 6.0 to 6.8, in contrast to the standard value of 4.1 for glutamic acid (Kuramitsu & Hamaguchi, 1980 ▶; Webb *et al.*, 2011 ▶). This abnormally high p*K*_a_ for Glu35 is believed to result from a hydrophobic environment mainly caused by Trp108 (Inoue *et al.*, 1992 ▶). It also provides a good benchmark for computer simulations of p*K*_a_ (Wallace & Shen, 2011 ▶; Goh *et al.*, 2014 ▶). Furthermore, Niimura *et al.* (1997 ▶) and Bon *et al.* (1999 ▶) studied the structure of lysozyme using neutron crystallography, which can observe H atoms. However, no structural arguments have yet appeared to explain the high p*K*_a_ of Glu35 consistently. In contrast, the present study of the high-pressure structure of lysozyme detects higher energy conformational states around Glu35 produced by pressure; these states cannot be detected in ambient crystallographic environments.

We have also observed a novel pressure-induced phase transition of the tetragonal crystal of HEWL. A disorder–order transition arising from pressure has previously been reported for Cowpea mosaic virus by Fourme *et al.* (2002 ▶). To the best of our knowledge, however, this is the first report of a pressure-induced crystal-to-crystal phase transition observed in a protein crystal.

## 2. Materials and methods   

### 2.1. Crystallization   

For this study, we used tetragonal crystals (space group *P*4_3_2_1_2) because their higher symmetry accommodates the limitations imposed on the oscillation angle by the aperture of the DAC, which was approximately 70° for the entrance and exit apertures for the X-ray beams. Tetragonal crystals of HEWL (Wako Pure Chemical Industries, Osaka, Japan) were obtained by the batch crystallization method from a solution consisting of 40 mg ml^−1^ lysozyme, 50 m*M* sodium acetate buffer pH 4.5, 0.8 *M* sodium chloride at 293 K. In order to discuss the effects of substrate binding on the behaviour of Glu35, a complex of HEWL with tetra-*N*-acetylchitotetraose [(GlcNAc)_4_; Dextra Laboratories, Berkshire, England] was also crystallized from 25 mg ml^−1^ lysozyme with a protein:substrate ratio of 1:1.2 and the same buffer conditions as the free enzyme. Because the solubility of tetragonal crystals of lysozyme increases with pressure (Suzuki *et al.*, 2002 ▶), the salt concentration of the solution was increased to 1.5 *M* before pressurization.

### 2.2. High-pressure protein crystallography experiment   

HPPX experiments were performed on beamline AR-NW12A at the Photon Factory. To collect high-completeness data sets at each pressure, we inserted two or three crystals into the DAC at different orientations. To prevent the crystals from moving in the DAC sample chamber, the crystals were fixed with tied fibres from cigarette filters (Nagae *et al.*, 2012 ▶). The pressure in the DAC was gradually increased to the target pressure in about 10–15 min, and the DAC was left for about 10 min. The pressure was then measured before and after each X-ray measurement using the wavelength shift of ruby fluorescence. Diffraction data sets were collected from HEWL crystals at room temperature at pressures of up to 950 MPa. The crystals diffracted reasonably well at pressures as high as 950 MPa, but no diffraction was detected at 1 GPa. The three-dimensional crystal structures were determined at a resolution of 1.5–1.9 Å. Data collection at atmospheric pressure was performed with a Rigaku FR-E Cu *K*α X-ray source and a R-AXIS VII detector using a glass capillary. Table 1 ▶ summarizes the data-collection and scaling statistics for all diffraction data sets.

### 2.3. Structure analysis   

The diffraction patterns were indexed, integrated and scaled using *HKL*-2000 (Otwinowski & Minor, 1997 ▶). During the integration, the mosaicity was used to check for radiation damage to the crystals. Below 890 MPa, the mosaicities before exposure were less than approximately 0.05°. At 950 MPa, however, the mosaicity increased to approximately 0.6° because a crystal-to-crystal phase transition occurs between 890 and 950 MPa, as discussed below. The structural analysis was based only on frames acquired at the lower mosaicity. The initial crystal structure of HEWL at 0.1 MPa (atmospheric pressure) was solved using *MOLREP* (Vagin & Teplyakov, 2010 ▶) with PDB entry 2lyz (Diamond, 1974 ▶) as the search model. For the complex with (GlcNAc)_4_, PDB entry 1lzc (Maenaka *et al.*, 1995 ▶) was used. The structure was refined using *REFMAC*5 (Murshudov *et al.*, 2011 ▶) implemented in the *CCP*4 suite (Winn *et al.*, 2011 ▶) and then by manual fitting with *Coot* (Emsley & Cowtan, 2004 ▶). The crystal structures of HEWL at different high pressures were solved using the structure at atmospheric pressure as the starting model, and were then improved by several rounds of refinement with *REFMAC*5 and *Coot*. At 950 MPa (*i.e.* after the phase transition), the space group changes and the crystals were merohedrally twinned. The structure solution and refinement were therefore performed on an untwinned data set using *DETWIN* in *CCP*4 (the twin fraction was 0.36). The four structures were also searched by *MOLREP* and refined with *REFMAC*5 and *Coot*. Water molecules were added using the solvent mode of *ARP*/*wARP* (Lamzin & Wilson, 1993 ▶). We removed water with *B* factors greater than 60 Å^2^, map root-mean-square deviation (r.m.s.d.) levels less than 1.00 e Å^−3^ and closest contacts less than 2.30 Å or greater than 3.50 Å. The refinement statistics are compiled in Table 2 ▶.

The solvent-excluded volumes of HEWL were determined using *VOIDOO* with a probe radius of 1.4 Å (Kleywegt & Jones, 1994 ▶). The volumes of the internal cavities were calculated using *CASTp* with a probe radius of 0.9 Å (Dundas *et al.*, 2006 ▶). The figures were generated using the *PyMOL* visualization software (DeLano, 2002 ▶). The displacement vectors shown in Figs. 2 and 4 were generated using the modevector.py script from the PyMOLWiki website (http://www.pymolwiki.org) with a 0.5 Å cutoff, and the difference-distance maps were calculated using *CMView* (Vehlow *et al.*, 2011 ▶). The internal cavities in Figs. 11 and 12 were generated using the *HOLLOW* tool (Ho & Gruswitz, 2008 ▶). Finally, the secondary-structure assignments of HEWL used in this paper are based on UniProt (http://www.uniprot.org/uniprot/P00698).

## 3. Results   

### 3.1. Compressibility of the unit cell and the HEWL molecule   

**Figure 1:**
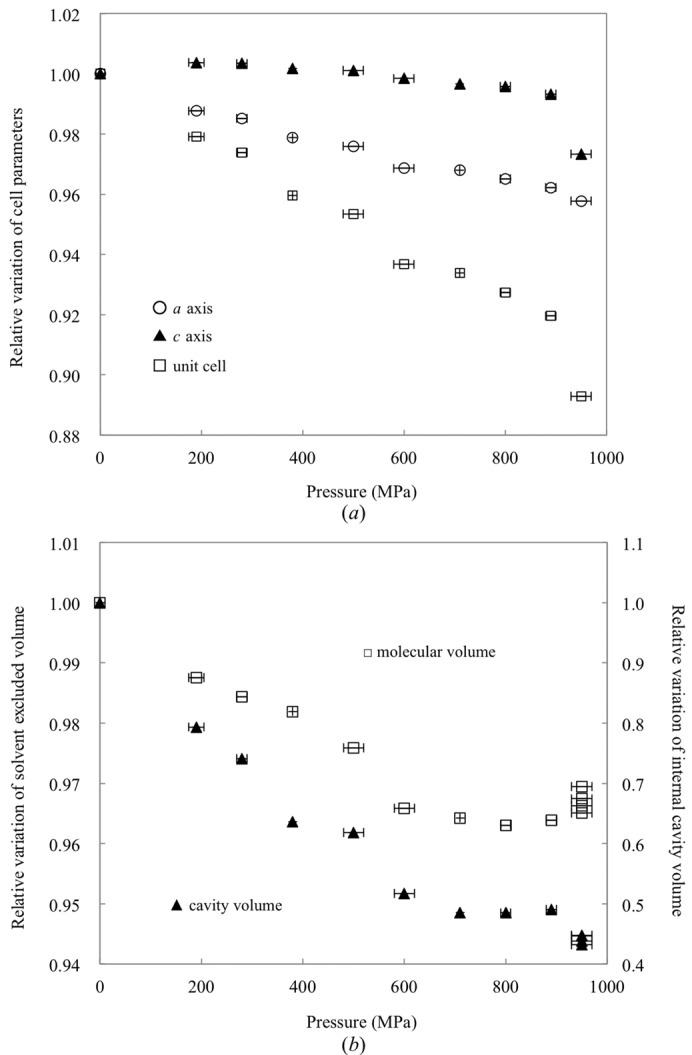
Relative variations of unit-cell parameters, volumes of molecules and volumes of internal cavities. (*a*) Unit-cell parameters *a* (empty circles) and *c* (filled triangles) and the unit-cell volume (empty squares) as a function of pressure. Values for 950 MPa were calculated in terms of the original *P*4_3_2_1_2 cell. (*b*) Relative variation in external solvent-excluded molecular volume (empty squares) and volume of internal cavities (filled triangles) of HEWL as a function of pressure.

Fig. 1 ▶(*a*) shows the relative variations in the unit-cell parameters and the unit-cell volume as a function of pressure. The unit-cell volume decreases from 2.38 × 10^6^ Å^3^ at atmospheric pressure to 2.19 × 10^6^ Å^3^ at 890 MPa. The compressibility of the unit cell is 9.4 × 10^−2^ GPa^−1^, which is almost identical to the values of 9.8 × 10^−2^ GPa^−1^ reported by Fourme *et al.* (2001 ▶) and 9.4 × 10^−2^ GPa^−1^ reported by Ascone, Savino *et al.* (2010 ▶). As previously reported by Fourme *et al.* (2001 ▶), the compression of the unit cell is anisotropic. The *a* axis decreases in size much more than the *c* axis. The compressibility of the *a* axis is 4.6 × 10^−2^ GPa^−1^, whereas the length of the *c* axis remains almost constant.

The molecular and cavity volumes of HEWL are 2.49 × 10^5^ and 7.48 × 10^3^ Å^3^ at atmospheric pressure and 2.40 × 10^5^ and 3.67 × 10^3^ Å^3^ at 890 MPa, respectively (Fig. 1 ▶ *b*). Both the molecular and cavity volumes decrease approximately linearly with pressure up to 710 MPa. The compressibility of the molecular volume is 4.8 × 10^−2^ GPa^−1^, which is almost equal to the published value of 4.7 × 10^−2^ GPa^−1^ (Kundrot & Richards, 1987 ▶), and the compressibility of the cavity volume is 0.80 × 10^−2^ GPa^−1^. However, from 710 to 890 MPa, which is just below the pressure of the phase transition, the molecular and cavity volumes remain almost constant.

### 3.2. Pressure-induced structural changes in molecules   

**Figure 2:**
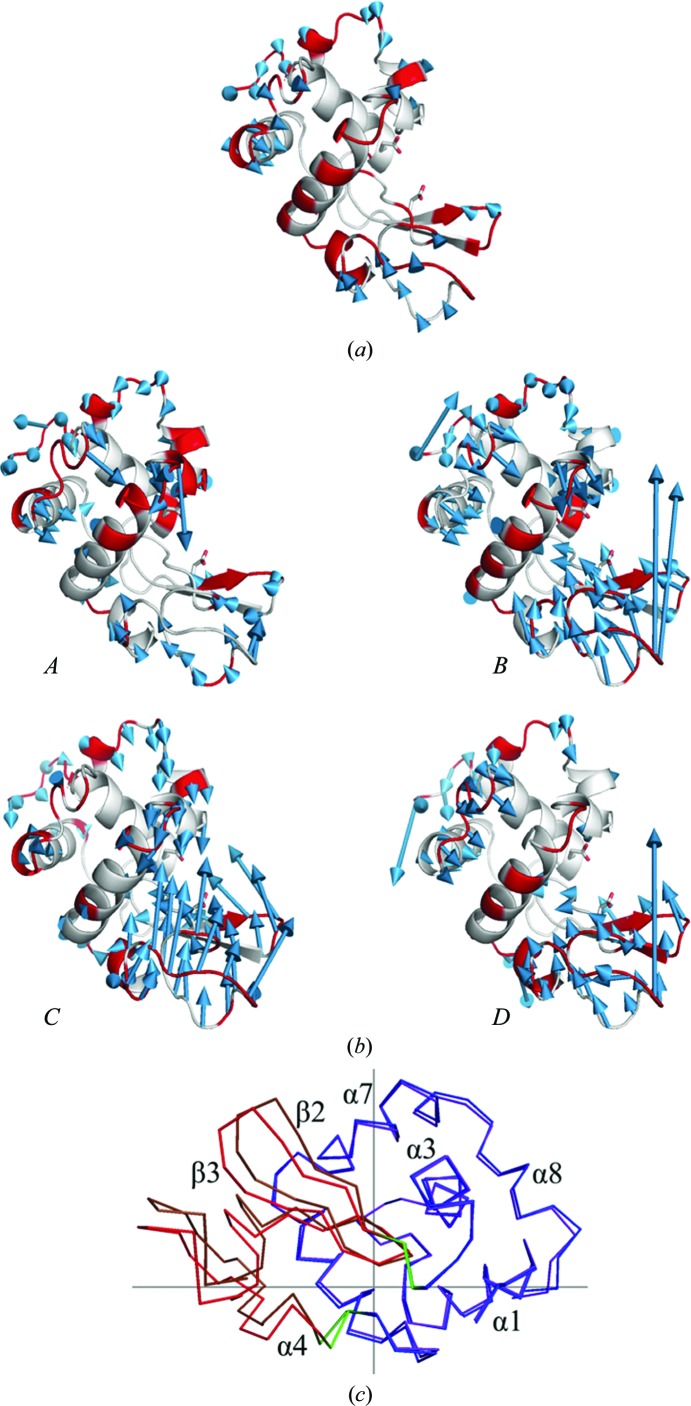
Pressure-induced changes in the structure of HEWL with respect to the structure at 0.1 MPa: (*a*) 890 MPa, (*b*) 950 MPa. For each structure, the arrows indicate the displacement vectors, which show the shift in C^α^ positions between 0.1 MPa and the final pressure. For clarity, displacements larger than the threshold of 0.5 Å are drawn and the lengths of the displacement vectors are increased tenfold. The 890 MPa structure is distorted to close the active site and the distortions of molecules increase at 950 MPa. At 950 MPa, there are four molecules, *A*, *B*, *C* and *D*, in the asymmetric unit, and each is distorted differently as shown in this figure. The red colour in the cartoon model shows that the region has higher *B* factors than the average for each molecule. (*c*) Large motion detected between 0.1 MPa and molecule *C* at 950 MPa using *DynDom*. The cross indicates the centre of rotation. The domains of the structure at 0.1 MPa are shown in blue (Lys1–Thr40, Leu83–Leu129) and red (Thr43–Ser81) and the corresponding domains at 950 MPa are shown in purple and brown, respectively. The residues in green (Gln41–Ala42, Ala82–Leu83) provide the hinges for the movement.

**Figure 3:**
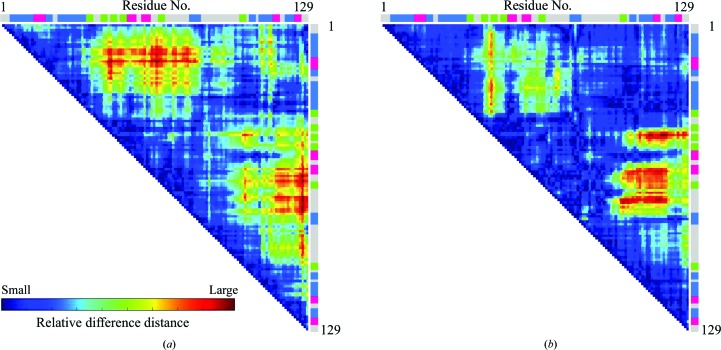
Difference-distance plot for C^α^ atoms. (*a*) Relative difference between the structures at 0.1 and 890 MPa and (*b*) for molecule *C* at 950 MPa. Regions with relatively large conformational change are highlighted in red, whereas regions that remain unchanged are shown in blue. Colour bars at the side and top indicate the secondary structures of HEWL assigned by UniProt (helices are blue, turns are red and sheets are green).

Fig. 2 ▶ shows the structural changes of the molecule at 890 and 950 MPa from the structure at ambient pressure. Each structure was superimposed based on the C^α^ atoms of all residues 1–129, and the r.m.s.d. was analyzed with the *CCP*4 program *SUPERPOSE* (Krissinel & Henrick, 2004 ▶). Under high pressures, the structures become distorted with respect to the structure at ambient pressure. At 890 MPa, the β-strand domain (residues 42–82) and the α-helix domain (residues 1–41 and 83–129) move towards each other to close the active site (Fig. 3 ▶ *a*).

**Figure 4:**
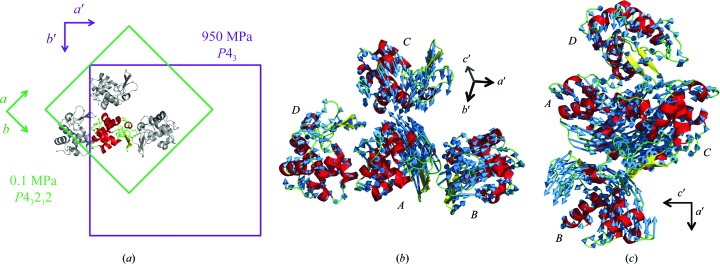
Crystal-to-crystal phase transition observed between 890 and 950 MPa. (*a*) Change in the unit cell associated with the transition. The space group changes from *P*4_3_2_1_2 to *P*4_3_. Four molecules are included in the asymmetric unit of the new *P*4_3_ cell, whereas the original unit cell contains one molecule in the asymmetric unit. The molecule corresponding to that contained in the asymmetric unit of the original *P*4_3_2_1_2 cell is colour-coded according to secondary structure and other three molecules are shown in grey. (*b*, *c*) General displacement of molecules after phase transition from the four corresponding molecules at 0.1 MPa viewed from two different directions. The arrows are the vectors between the C^α^ positions of the two structures. Molecules *A* and *C* approach each other along the *c* axis. To facilitate visualization, displacements larger than the threshold of 0.5 Å are drawn and the lengths of the vectors are magnified threefold.

Beyond this pressure range, a crystal-to-crystal phase transition occurs (Fig. 4 ▶). At 950 MPa, the crystal symmetry decreases with respect to that at lower pressure, and the space group changes from *P*4_3_2_1_2 to *P*4_3_. The *a* axis of the new *P*4_3_ crystal corresponds to the diagonal (*a* + *b*) of the original *P*4_3_2_1_2 crystal lattice. Four molecules (*A*, *B*, *C* and *D*) are included in the asymmetric unit of the new *P*4_3_ crystal, whereas these four molecules are symmetry mates in the original (low-pressure) *P*4_3_2_1_2 crystal, which has one molecule in the asymmetric unit. Figs. 4 ▶(*b*) and 4 ▶(*c*) show the general displacement of molecules on going from 0.1 to 950 MPa. The results also indicate that when the unit-cell volume is calculated in terms of the original *P*4_3_2_1_2 unit cell, its volume dramatically decreases after the phase transition (Fig. 1 ▶ *a*). In particular, the *c* axis is significantly shortened. We estimate the average r.m.s.d. of the C^α^-atom positions between 0.1 and 890 MPa to be 0.42 Å. However, the corresponding r.m.s.d.s for molecules *A*, *B*, *C* and *D* after the phase transition (at 950 MPa) are estimated to be 0.57, 0.70, 0.72 and 0.63 Å, respectively. One of the four molecules in the asymmetric unit, molecule *C*, is dramatically more distorted, and the active-site groove becomes significantly more closed (Figs. 2 ▶ *b* and 3 ▶ *b*). We analyzed the domain movement of molecule *C* between 0.1 and 950 MPa using *DynDom* (Hayward & Berendsen, 1998 ▶). The hinge regions of this movement are residues 41 and 42 and residues 82 and 83 (coloured green in Fig. 2 ▶ *c*). The former occurs between *β*1 and *β*2 and the latter occurs in the middle of α4. The degree of closure motion is 59.2%. The rotational angle and conformational translation between the α-domain and the β-domain of the high-pressure (950 MPa) molecule *C* *versus* the ambient (0.1 MPa) structure are approximately 6.5° and 0.5 Å, respectively.

### 3.3. Reduction in temperature factor   

**Figure 5:**
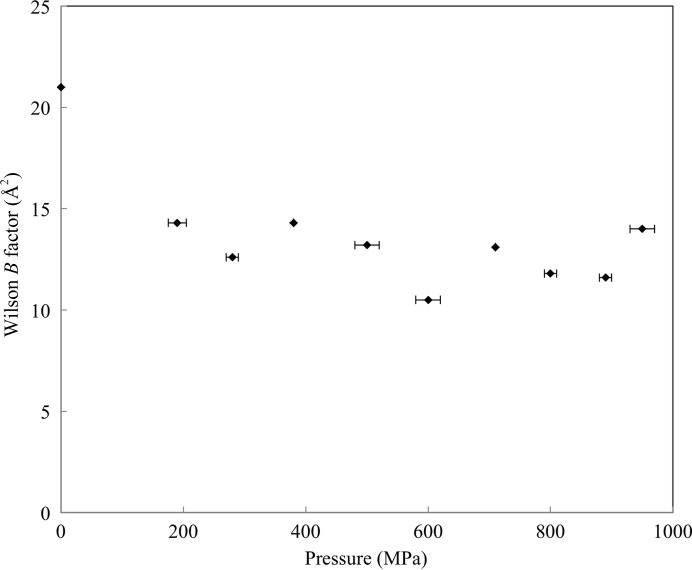
The Wilson *B* factor as a function of pressure.

**Figure 6:**
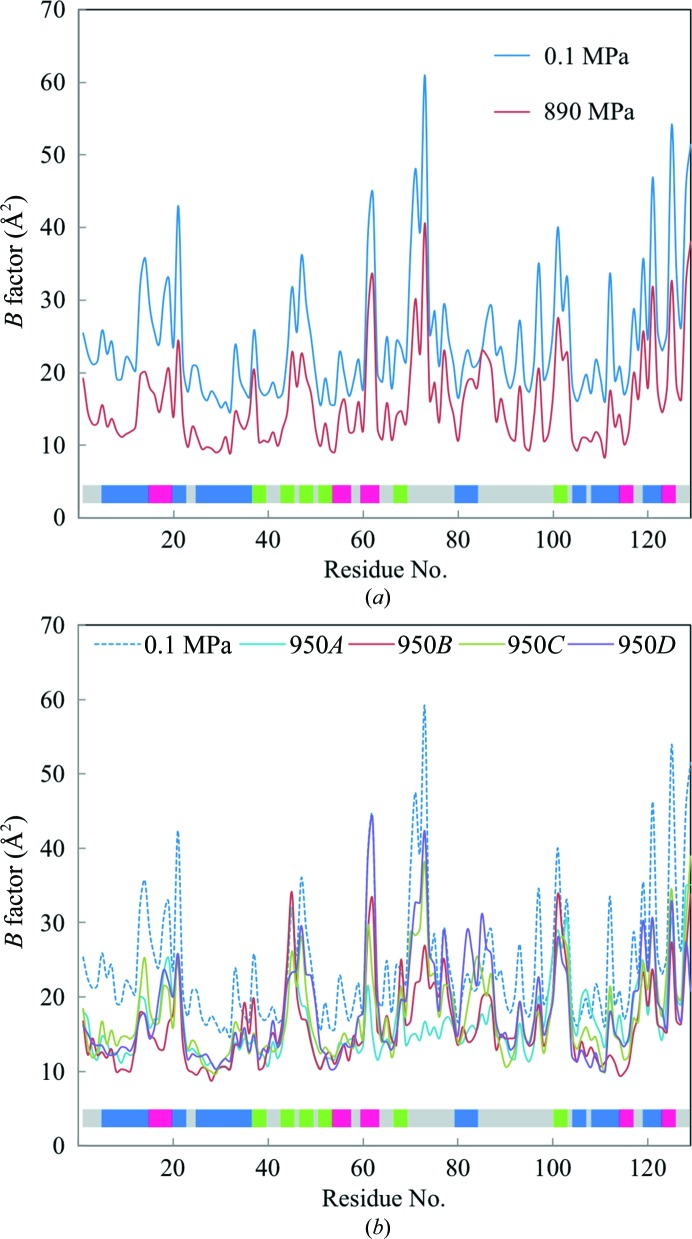
Average *B* factor of all atoms as a function of residue number. (*a*) *B* factors at 0.1 MPa (blue line) and 890 MPa (red line) and (*b*) *B* factors at 0.1 MPa (blue dashed line) and 950 MPa for molecules *A*, *B*, *C* and *D* (cyan, red, green and purple lines, respectively). The colour bar indicates the secondary structures of HEWL assigned by UniProt (helices are blue, turns are red and sheets are green).

As shown in Fig. 5 ▶, the Wilson *B* factors are reduced upon increasing the pressure from 0.1 to 890 MPa. In particular, the reduction between 0.1 to 190 MPa is significant. This initial decrease is also observed in the molecular and cavity volume reduction shown in Fig. 2 ▶. Fig. 6 ▶ shows the average temperature factors for all atoms in each residue at 0.1, 890 and 950 MPa. At 890 MPa, the decrease in the average *B* factor is uniform for the overall structure. At 950 MPa, despite the lower resolution than at 0.1–890 MPa, the average *B* factor also decreases across the molecules. However, the *B* factors around β2–β3 and β6 do not decrease with respect to their values at 0.1 MPa.

### 3.4. Changes in hydration structure   

#### 3.4.1. Molecule surface   

**Figure 7:**
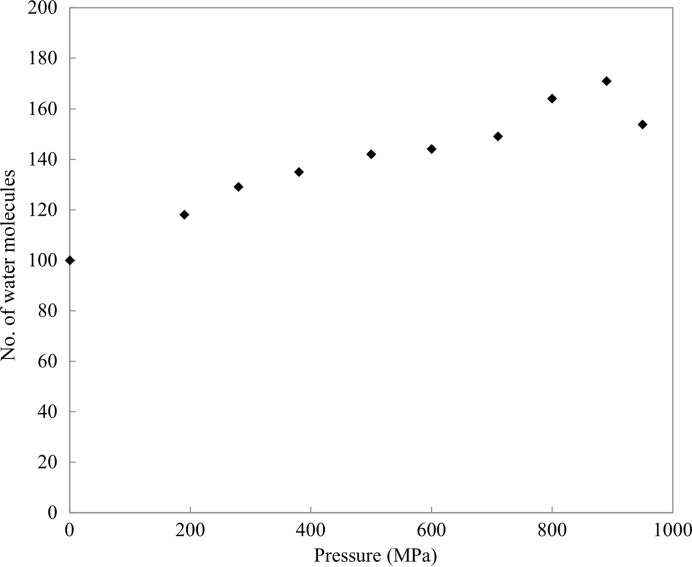
Number of water molecules as a function of pressure. At 950 MPa, the number of water molecules is the average over the four molecules in the asymmetric unit.

**Figure 8:**
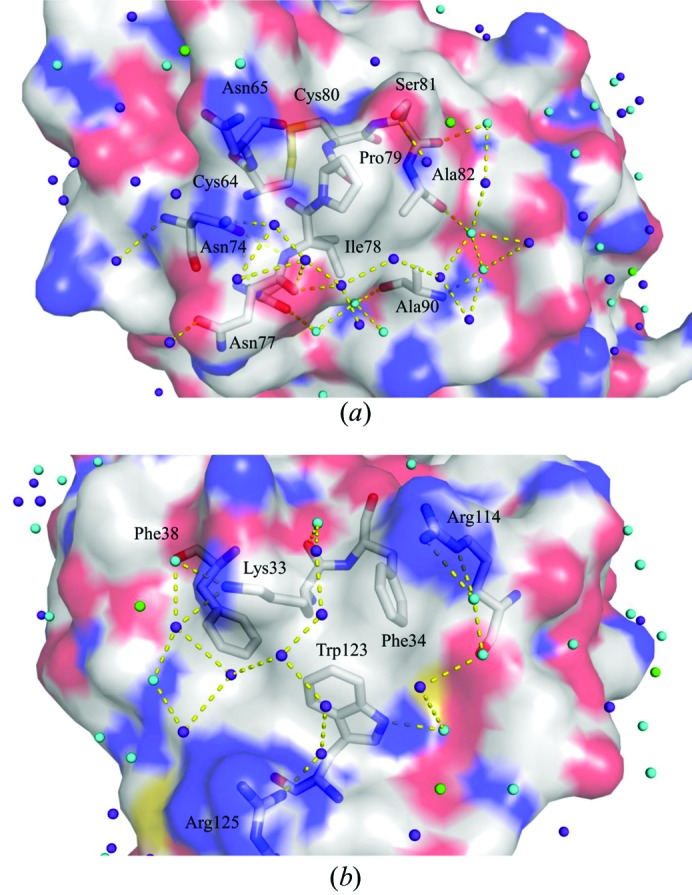
Water molecules observed on the hydrophobic surface of HEWL at 890 MPa. (*a*) Hydration structure around Ile78 and Pro79. (*b*) Hydration structure around Phe34, Phe38 and Trp123. Upon increasing the pressure, several water molecules appear and form a hydrogen-bond network with each other over the hydrophobic surface. The surface in red is negatively charged, that in blue is positively charged and that in white is neutral. Water molecules found only at 0.1 MPa are coloured green, those found only at 890 MPa are coloured purple and those found at both 0.1 and 890 MPa are coloured cyan.

**Figure 9:**
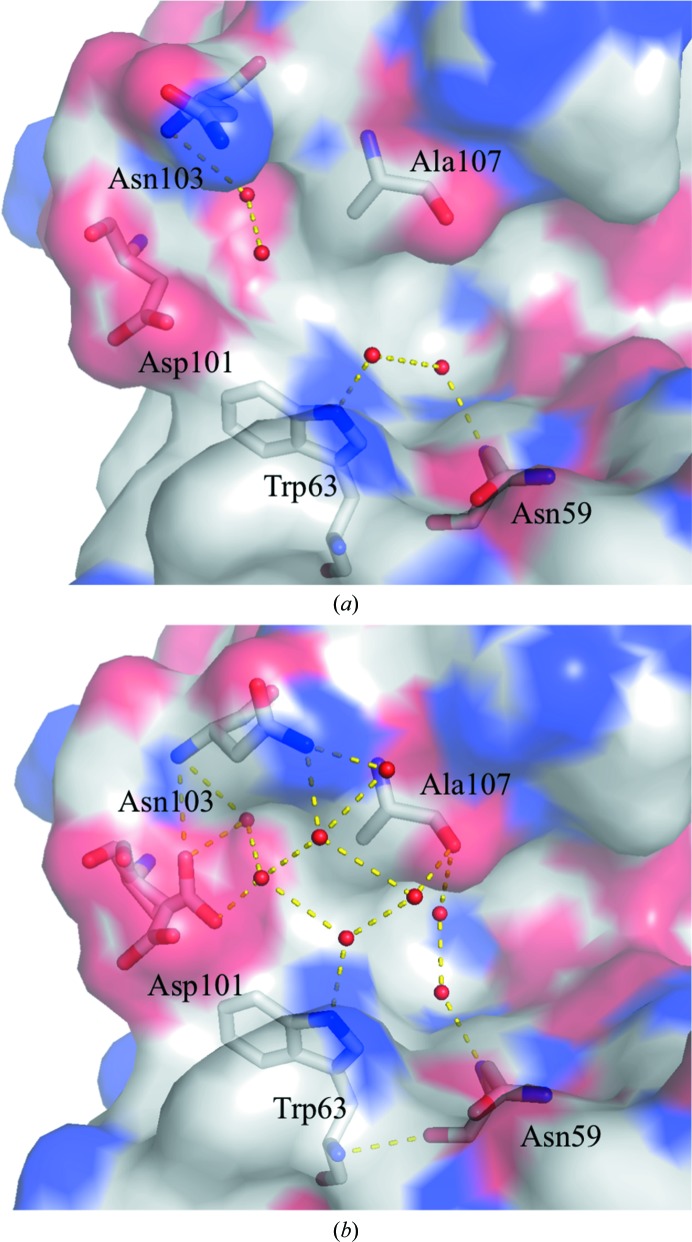
Change in the structure of HEWL upon hydration on going from 0.1 to 890 MPa: (*a*) 0.1 MPa and (*b*) 890 MPa. The conformation of Asn103 changes to hydrogen-bond to a newly arrived water molecule at 890 MPa. The protein surface is coloured as in Fig. 11 ▶.

The number of water molecules observed gradually increases with pressure. For example, we assign 171 water molecules at 890 MPa compared with 100 at 0.1 MPa (Fig. 7 ▶). Among these water structures, 81 water molecules are conserved at both 0.1 and 890 MPa, and 90 new water molecules are found at 890 MPa, mostly located at the back surface of the active site. In addition, hydration develops on a hydrophobic surface of the protein, supported by hydrogen-bond networks. Examples of such water structures appear beside the hydrophobic residues Ile78 and Pro79 and Phe34, Phe38 and Trp123 (Fig. 8 ▶). The newly incorporated water molecules hydrogen-bond to protein residues or to the original water molecules that were present at 0.1 MPa. Side-chain conformational changes also help to anchor the network of new water molecules. For example, at 890 MPa, the side-chain conformations of Asn103 are modified to allow hydrogen-bonding to two new water molecules (Fig. 9 ▶). However, the number of observed water molecules decreases again at 950 MPa (Fig. 7 ▶), which may reflect an increase in disorder caused by the large movements incurred during the pressure-induced phase transition. This interpretation is also supported by the lower resolution of the diffraction data at 950 MPa.

#### 3.4.2. Internal cavity   

**Figure 11:**
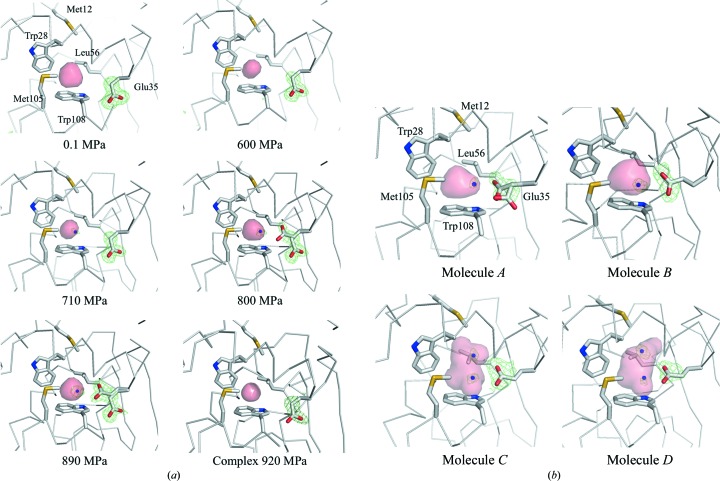
Penetration of water and change in conformation of Glu35 in the internal cavity near Trp108: (*a*) from 0.1 to 890 MPa and at 920 MPa for the (GlcNAc)_4_ complex and (*b*) for the four molecules at 950 MPa. The internal cavity is represented as a pink surface. The green mesh shows the difference electron-density map calculated by omitting the water that penetrates and a Glu35 side chain. Maps are contoured at (*a*) 3.0σ and (*b*) 2.5σ.

**Figure 12:**
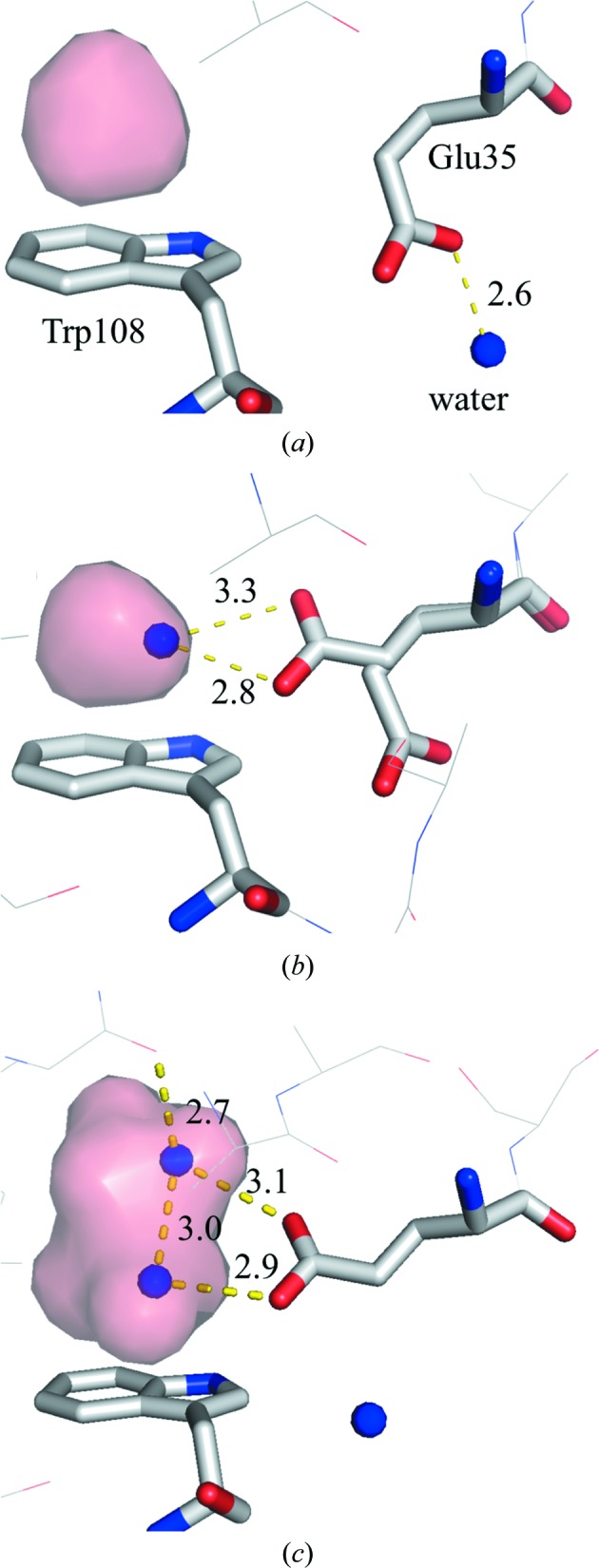
Magnified view near the cavity over Trp108: (*a*) 0.1 MPa, (*b*) 890 MPa and (*c*) molecule* C* of the 950 MPa structure. Residues surrounding the hydrophobic cavity, including Glu35 and Trp108, are represented as sticks. The internal cavity is represented as a pink surface. The water molecules that penetrate are represented by blue balls. Hydrogen bonds are illustrated by yellow dashed lines.

As the pressure increases, most of the internal cavities are compressed (Figs. 1 ▶ *b* and 10 ▶). However, the volume of the cavity above Trp108 does not decrease monotonically with pressure. Instead, the volume of this cavity initially decreases from 0.1 to 600 MPa (with volumes of 62.5, 49.6, 48.2, 46.8 and 39.5 Å^3^ at pressures of 0.1, 190, 380, 500 and 600 MPa, respectively). The cavity then swells to 44.6 Å^3^ at 710 MPa and is compressed again to 42.4 and 43.0 Å^3^ at 800 and 890 MPa, respectively. This increase in cavity volume comes from water penetrating into the cavity and from the movement of the surrounding residues. In fact, at pressures greater than 710 MPa a water molecule penetrates into the cavity (Fig. 11 ▶ *a*). This water molecule appears to interact with Trp108 *via* a lone pair–π interaction (Egli & Sarkhel, 2007 ▶; Jain *et al.*, 2009 ▶). Upon increasing the pressure to 800 MPa, Glu35 becomes a dual conformer. The side chain of the new conformer is inserted into the cavity so that it hydrogen-bonds to the penetrating water molecule.

Above the phase-transition pressure, Glu35 of the four molecules adopts different conformations (Fig. 11 ▶ *b*). In molecule *A* Glu35 takes on dual conformations, as in the 890 MPa structure. In molecules *B*, *C* and *D*, however, the side chains have a single conformation and protrude into the cavity to hydrogen-bond to the newly present water molecules. In molecules *C* and *D* additional water molecules penetrate above the previously present water molecule that interact with Trp108 and make a hydrogen-bond network between Trp108 and the main-chain carbonyl O atom of Trp28.

However, in the case of the complex of HEWL with (GlcNAc)_4_, the side chain of Glu35 is only in the outward conformation at 920 MPa and no water penetration into the cavity above Trp108 was observed, as shown in Fig. 11 ▶(*a*). A phase transition did not take place even when the crystal was pressurized to 920 MPa.

## 4. Discussion   

### 4.1. Pressure-induced phase transition   

In the solution state, the HEWL molecule is expected to be denatured at pressures of several hundred MPa (Heremans & Wong, 1985 ▶; Jonas, 1990 ▶). In the crystal form, however, the HEWL molecule can tolerate pressures of up to 950 MPa. As previously mentioned by Katrusiak & Dauter (1996 ▶), the crystal packing might mitigate the effects of pressure. Upon increasing the pressure, a pressure-induced phase transition is observed between 890 and 950 MPa. This result is consistent with a previous HPPX study of HEWL, which reported a radical reduction of the unit-cell volume, diffuse scattering and the absence of diffraction after 15 min at 915 MPa (Fourme *et al.*, 2001 ▶). This result of Fourme and coworkers may thus be the onset of the phase transition. At the phase transition, the four molecules in the asymmetric unit move towards each other (Fig. 4 ▶). In particular, molecules *A* and *C* approach each other along the *c* axis. Consequently, the *c* axis may become shorter, as shown in Fig. 1 ▶(*a*). After the phase transition, each structure in the asymmetric unit may embody slightly different high-energy conformations of HEWL, which are difficult to observe using the usual crystallographic methods because of their relatively low abundance at atmospheric pressure. As pointed out by Collins *et al.* (2011 ▶) and Fourme *et al.* (2012 ▶), such structures are often essential for protein function. We discuss these aspects below.

### 4.2. Change in the volume and the structure of HEWL   

In accordance with Le Chatelier’s principle, an increase in pressure favours the reduction of the volume of a system. As shown in Fig. 1 ▶(*a*), the unit-cell volume of tetragonal crystals of HEWL decreases monotonically upon increasing the pressure. Note, however, that the molecular volume and the internal-cavity volume are almost constant from 710 to 890 MPa (Figs. 1 ▶ *b* and 11 ▶ *a*). At 710 MPa, the unit-cell and molecular volumes are compressed to 93.4 and 96.4% with respect to the corresponding volumes at atmospheric pressure. This is consistent with previous reports of the unit cell exhibiting a compressibility larger than that of the molecules (Ascone, Kahn *et al.*, 2010 ▶; Refaee *et al.*, 2003 ▶). The partial molar volume (PMV) of a protein is expressed by the sum of the volumes of all the constituent atoms, the volumes of void cavities and the change in volume because of hydration. As viewed from the perspective of the PMV, the system shrinks in going from atmospheric pressure to 710 MPa by compressing the volume of the internal cavities. At pressures greater than 710 MPa, however, the system reduces the PMV mainly by filling the cavity above Trp108 with a water molecule from the bulk region (Fig. 11 ▶). This seems to explain why the molecular and internal-cavity volumes are outwardly constant from 710 to 890 MPa, whereas the unit-cell volume decreases monotonically. The same trend occurs in 3-isopropylmalate dehydrogenase from *Shewanella oneidensis* MR-1 (SoIPMDH; Nagae *et al.*, 2012 ▶). This phenomenon might depend on the original size of the cavity in question. At 0.1 MPa, the volume of the cavity above Trp108 is 62.5 Å^3^ and that at the dimer interface of SoIPMDH is 55.6 Å^3^. While, on the other hand, the hydrophobic cavity of the L99A mutant of T4 lysozyme is large as 160 Å^3^ (Collins *et al.*, 2005 ▶). In this case, the cavity is sufficiently large to accommodate water molecules, so its volume need not increase.

Despite the disadvantage of water localization in the hydrophobic environment, this penetration by water may be the main way to reduce the PMV of the molecule when the compression of the cavities reaches a physical limit. In studies involving a three-dimensional reference interaction-site model (RISM) theory of molecular solvation, Imai *et al.* (2007 ▶) reported that reduction of the PMV is associated with a pressure-induced structural change in ubiquitin. They attribute the reduction of the PMV primarily to the penetration of water into the hydrophobic core of ubiquitin. Imai & Sugita (2010 ▶) subsequently used a molecular-dynamics (MD) simulation to confirm the pressure-induced conformational changes linked to penetration by water. In lysozyme, to cancel out the drawback of water localization in a hydrophobic environment, the water that penetrates the cavity forms a lone pair–π interaction with the indole ring of the Trp108 side chain (Figs. 11 ▶ and 12 ▶). At pressures greater than 800 MPa, the side chain of the active residue Glu35 becomes a dual conformer. This emerging conformer may also reduce the PMV of the molecule and be stabilized by hydrogen bonds to the water molecules that have penetrated the cavity.

Regarding the pressured-induced structural changes of HEWL, Kundrot & Richards (1987 ▶) previously reported a difference-distance map between the structure at ambient pressure and at 100 MPa, which indicates that with increasing pressure the distance between the β-domains and the α-domains decreases. These results provide evidence for a general tendency of the protein to become concentrated closer to its centre of mass, although some differences in directions persist. This tendency to bring the β-domains and α-domains into proximity corresponds to our results at 890 MPa (Figs. 2 ▶ *a* and 3 ▶ *a*). However, a large deformation of the β-domain is detected only for molecule *C* at 950 MPa. In particular, molecule *C* undergoes a relatively large twist and closure (Fig. 2 ▶ *c*), as detected by *DynDom*. This twist of the β-domain is consistent with the results of high-pressure NMR studies (Refaee *et al.*, 2003 ▶).

### 4.3. Pressure-induced change in the hydration structure at the surface of HEWL   

Previous HPPX studies of lysozyme, Cowpea mosaic virus and IPMDH have reported a greater number of water molecules in the high-pressure structures and a consequential increase in the number of hydrogen bonds between water molecules and protein (Kundrot & Richards, 1987 ▶; Girard *et al.*, 2005 ▶; Nagae *et al.*, 2012 ▶). The present results also indicate an increase in the number of water molecules upon pressurization (Fig. 7 ▶). Most new water molecules are localized on the surface of HEWL, which suggests that pressure suppresses atomic fluctuations, thereby reducing the PMV. Actually, the overall *B* factor decreases with pressure (by 8.7 Å^2^ on going from 0.1 to 890 MPa; Fig. 5 ▶). A pressure-induced decrease in the *B* factor was also revealed by structural studies of Cowpea mosaic virus (Girard *et al.*, 2005 ▶). MD simulations also indicate a pressure-induced decrease in atomic positional fluctuations (Brunne & van Gunsteren, 1993 ▶) and enhanced interactions between water molecules and protein (Marchi & Akasaka, 2001 ▶).

The present HPPX study reveals several water molecules on the hydrophobic surface of the protein forming a hydrogen-bond network between themselves (Figs. 8 ▶ and 9 ▶). Such localization may be representative of the conformational change of water molecules driven by the pressure-induced necessity to reduce the PMV. Some residues change their side-chain conformation to interact with structured water molecules and also to secure hydrogen-bond networks at hydrophobic surfaces.

### 4.4. Pressure-induced conformational change of Glu35   

As the pressure increases, Glu35, which is one of the two residues with a catalytic carboxyl group, changes its conformation. Between 0.1 and 600 MPa, Glu35 thrusts its side chain towards the active site. At 800 MPa, however, one of the dual conformers of Glu35 assumes a novel conformation in which its side chain faces an inner side of the hydrophobic cavity above Trp108. At higher pressure, for example in the structures of molecules *B*, *C* and *D* at 950 MPa, only the inward conformation is observed (Fig. 11 ▶ *b*). The p*K*_a_ of this glutamic acid is known to be 6.0–6.8, which is abnormally high (Kuramitsu *et al.*, 1977 ▶; Webb *et al.*, 2011 ▶) compared with the standard p*K*_a_ of glutamic acid (4.1). This high p*K*_a_ of Glu35 is critical for it to serve as a general acid catalyst in the glycosidase activity of lysozyme under physiological conditions. The hydrophobicity of Trp108 is considered to contribute to the high p*K*_a_ of Glu35. Single mutations of Trp108 to the less hydrophobic residues Tyr and Gln decrease the activity of HEWL, and the p*K*_a_ of Glu35 is reduced by 0.2 and 0.6, respectively (Inoue *et al.*, 1992 ▶). To the best of our knowledge, these inward conformations have not yet been observed in crystalline lysozyme, including in neutron diffraction experiments (Niimura *et al.*, 1997 ▶; Bon *et al.*, 1999 ▶).

We used the empirical methods *DEPTH* (Tan *et al.*, 2013 ▶) and *PROPKA* (Søndergaard *et al.*, 2011 ▶) to estimate the p*K*_a_ of Glu35 in the structures determined by HPPX. As expected, the p*K*_a_ values of the inward conformation are greater than those of the outward conformation (Table 3 ▶). Using *DEPTH*, the p*K*_a_ values of the inward Glu35 are estimated to range from 6.3 to 6.5, which is consistent with the experimental results, whereas those of the outer conformation are less than 4.5. *PROPKA* also assigns greater p*K*_a_ for the inward conformation than for the outward conformation, but the p*K*_a_ of the glutamine side chain seems to be overestimated.

In an interesting twist, the refined NMR structure of HEWL shows that the inward or buried conformation of Glu35 is popular in solution (PDB entry 1e8l; Schwalbe *et al.*, 2001 ▶). Surprisingly, 45 of the 50 low-energy structures include the inward conformer of Glu35. Thus, the inward conformation of Glu35 appears to be common in the solution state. Why conventional crystallography does not detect the inward conformation of the NMR structure at ambient pressure is not clear. Between NMR and X-ray structures, the root-mean-square deviation around Glu35 is not high; it is less than 1.0 Å (Schwalbe *et al.*, 2001 ▶). One likely explanation invokes differences in salt concentrations and molecular packing in the crystal. Whether rightly or wrongly, the structure as determined by NMR does not explain why the side chain of Glu35 prefers an inward conformation. The HPPX results of this study show that water molecules penetrate into the hydrophobic cavity, thereby stabilizing the inward conformation of Glu35 by hydrogen-bonding to it. These results show that Trp108 is important not only for its hydrophobicity, but also for its ability to stabilize the water molecule in the hydrophobic cavity *via* a lone pair–π interaction.

However, the inward conformation of Glu35 and water penetration above Trp108 were never observed in the crystal complexed with (GlcNAc)_4_ even at 920 MPa. Pressure-induced phase transition with a large displacement of the molecule is not also observed for the complex crystal. These results show that the flexibility of the HEWL molecule is restricted when substrate is bound to the enzyme molecule. The inward conformation of Glu35 might occur only when the HEWL molecule is free from substrate. Thus, the present study shows that applying high pressure to crystallized HEWL changes the populations of the conformational substates, thereby revealing structures that are less prevalent at atmospheric pressure in the crystal (Collins *et al.*, 2011 ▶; Fourme *et al.*, 2012 ▶). Studies of these conformational substates can yield valuable information about the activity of proteins, for example, in the catalytic mechanism of enzymes.

## Supplementary Material

PDB reference: HEWL, 0.1 MPa, 4wld

PDB reference: 190 MPa, 4wlt

PDB reference: 280 MPa, 4wlx

PDB reference: 380 MPa, 4wly

PDB reference: 500 MPa, 4wm1

PDB reference: 600 MPa, 4wm2

PDB reference: 710 MPa, 4wm3

PDB reference: 800 MPa, 4wm4

PDB reference: 890 MPa, 4wm5

PDB reference: 950 MPa, 4wm6

PDB reference: complex with (GlcNAc)_4_, 920 MPa, 4xen

## Figures and Tables

**Figure 10:**
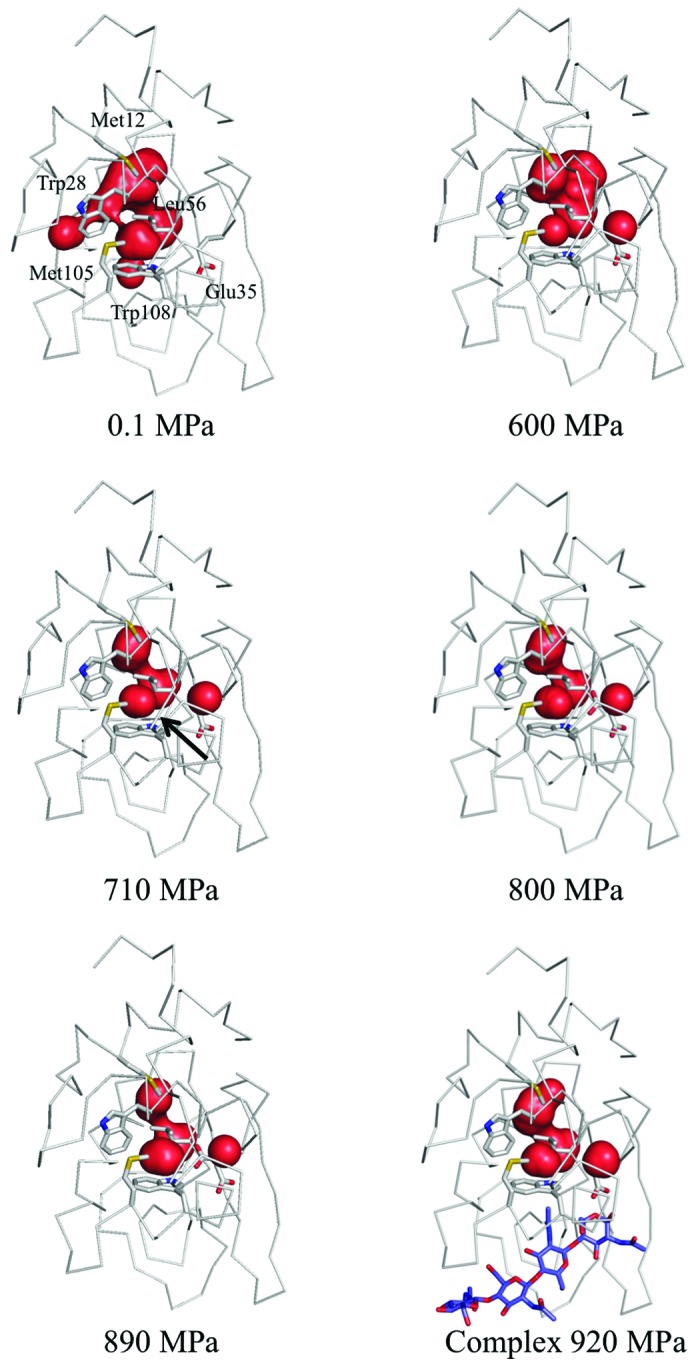
Internal cavities of HEWL from 0.1 to 890 MPa and at 920 MPa for the (GlcNAc)_4_ complex. Most of the cavities are compressed as the pressure increases. The cavity for which the volume increases upon penetration by water is indicated by a black arrow in the 710 MPa structure. The bound (GlcNAc)_4_ is coloured purple.

**Table 1:** Data-collection and processing statistics Values in parentheses are for the outer shell.

PDB code	4wld	4wlt	4wlx	4wly	4wm1	4wm2	4wm3	4wm4	4wm5	4wm6	4xen
Pressure (MPa)	0.1	190	280	380	500	600	710	800	890	950	920
Diffraction source	Rigaku FR-E	NW12A, PF-AR									
Wavelength ()	1.54	0.75	0.75	0.71	0.75	0.75	0.71	0.75	0.71	0.71	0.75
Temperature	Room temperature										
Detector	R-AXIS VII	ADSC Quantum 210r									
Crystal-to-detector distance (mm)	70	200	200	200	200	200	200	200	200	200	200
Rotation range per frame ()	1	1	1	1	1	1	1	1	1	1	1
Exposure time per frame (s)	30	1	1	5	5	1	1	1	5	3	2
No. of crystals used	3	3	2	2	2	2	2	2	2	3	2
Space group	*P*4_3_2_1_2	*P*4_3_2_1_2	*P*4_3_2_1_2	*P*4_3_2_1_2	*P*4_3_2_1_2	*P*4_3_2_1_2	*P*4_3_2_1_2	*P*4_3_2_1_2	*P*4_3_2_1_2	*P*4_3_	*P*4_3_2_1_2
Unit-cell parameters											
*a* ()	79.197	78.219	78.023	77.510	77.287	76.713	76.661	76.434	76.203	107.269	76.152
*c* ()	37.900	38.036	38.031	37.967	37.938	37.836	37.772	37.743	37.643	36.891	37.962
Mosaicity ()	0.350.37	0.040.09	0.040.06	0.040.06	0.050.21	0.040.06	0.040.08	0.040.08	0.030.15	0.350.54	0.200.40
Resolution range ()	50.001.54 (1.571.54)	50.001.60 (1.631.60)	50.001.60 (1.631.60)	50.001.62 (1.651.62)	50.001.60 (1.631.60)	50.001.60 (1.631.60)	50.001.55 (1.581.55)	50.001.60 (1.631.60)	50.001.60 (1.631.60)	50.001.85 (1.881.85)	50.001.55 (1.581.55)
Total No. of reflections	1450455	96458	998865	104019	75894	96701	112558	91102	89579	130892	122549
No. of unique reflections	18464	16148	16058	15293	15744	15483	16944	15331	15201	36471	16785
Completeness (%)	99.4 (99.9)	99.2 (99.9)	99.6 (100.0)	92.7 (95.0)	97.4 (99.5)	99.4 (99.6)	99.4 (100.0)	98.2 (99.3)	99.3 (100.0)	99.3 (99.5)	99.3 (98.9)
Multiplicity	7.8 (8.0)	6.0 (6.0)	6.2 (6.4)	7.6 (8.0)	5.0 (5.1)	6.3 (6.5)	6.7 (7.0)	6.1 (6.0)	5.9 (6.1)	4.0 (3.4)	7.4 (6.4)
*I*/(*I*)	47.3	46.4	51.5	50.3	46.2	52.9	48.9	49.0	39.1	19.1	44.5
*R*_merge_ † (%)	5.3 (38.8)	4.9 (32.3)	4.5 (24.8)	5.6 (38.6)	4.7 (20.1)	4.2 (15.2)	5.3 (35.9)	4.6 (22.4)	6.6 (38.2)	11.8 (53.3)	5.8 (28.6)

† *R*_merge_ is defined as 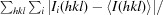 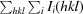, where *I*_*i*_(*hkl*) is the *i*th observation of reflection *hkl* and *I*(*hkl*) is the weighted mean of all observations (after rejection of outliers).

**Table 2:** Structure solution and refinement Values in parentheses are for the outer shell.

PDB code	4wld	4wlt	4wlx	4wly	4wm1	4wm2	4wm3	4wm4	4wm5	4wm6	4xen
Pressure (MPa)	0.1	190	280	380	500	600	710	800	890	950	920
No. of reflections, working set	16667	15188	15164	13435	14528	14593	15945	14276	14298	30495	15797
No. of reflections, test set	896	800	794	716	762	766	851	752	758	1606	839
*R*_work_ † (%)	14.51	15.30	15.18	15.45	16.01	15.53	18.14	16.76	16.30	19.92	16.40
*R*_free_ ‡ (%)	17.42	19.70	19.31	19.38	20.80	20.09	25.91	22.38	21.67	28.45	20.94
Cruickshank DPI	0.07	0.08	0.08	0.10	0.09	0.09	0.09	0.10	0.09	0.24	0.09
No. of non-H atoms											
Protein	1009	1031	1031	1031	1020	1025	1023	1032	1026	4013	1063
Ligand/ion	1	7	7	7	7	5	4	5	5	4	62
Water	100	118	129	135	142	144	149	164	171	615	172
Total	1110	1156	1167	1173	1169	1174	1176	1201	1202	4632	1297
*B* factor (^2^)											
Protein	25.14	18.79	16.99	19.32	17.63	14.65	16.76	15.74	16.28	17.38	11.92
Ligand/ion	39.51	37.17	34.76	34.14	29.83	27.80	22.17	29.27	28.95	29.56	23.32
Water	38.72	33.21	32.34	34.41	33.10	28.64	29.78	30.53	30.31	24.16	27.08
R.m.s.d. from ideality											
Bond lengths ()	0.024	0.024	0.024	0.024	0.025	0.022	0.022	0.025	0.023	0.015	0.021
Bond angles ()	2.166	2.151	2.146	2.099	2.157	2.132	2.132	2.011	2.036	1.728	2.202
Ramachandran plot											
Most favoured (%)	99	99	98	98	98	98	98	98	98	96	97
Allowed (%)	1	1	2	2	2	2	2	2	2	4	3

† *R*_work_ is defined as 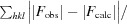 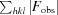.

‡ *R*_free_ is calculated using 5% of the data that were randomly chosen and excluded from the refinement.

**Table 3:** Estimated p*K*_a_ value of Glu35 in each conformation using *DEPTH* and *PROPKA*

	*DEPTH*		*PROPKA*	
Glu35 conformation	Outer	Inner	Outer	Inner
Pressure (MPa)	p*K*_a_	p*K*_a_	p*K*_a_	p*K*_a_
0.1	4.25		6.51	
190	4.37		6.62	
280	4.42		6.63	
380	4.36		6.67	
500	4.46		6.76	
600	4.15		6.80	
710	4.25		7.02	
800	4.09	6.34	6.84	10.97
890	4.08	6.48	6.73	10.00
950, molecule *A*	4.45	6.44	7.08	10.06
950, molecule *B*		6.32		10.19
950, molecule *C*		6.54		10.58
950, molecule *D*		7.97		9.76
PDB entry 2lzt	4.34		6.51	
